# Development and external validation of dual online tools for prognostic assessment in elderly patients with high-grade glioma: a comprehensive study using SEER and Chinese cohorts

**DOI:** 10.3389/fendo.2023.1307256

**Published:** 2023-11-21

**Authors:** Hongyu Zhang, Xinzhan Jiang, Fubin Ren, Qiang Gu, Jiahao Yao, Xinyu Wang, Shuhuai Zou, Yifan Gan, Jianheng Gu, Yongji Xu, Zhao Wang, Shuang Liu, Xuefeng Wang, Baojian Wei

**Affiliations:** ^1^ Department of Neurosurgery, The Fourth Affiliated Hospital of Harbin Medical University, Harbin, China; ^2^ Department of Neurobiology, Harbin Medical University, Harbin, China; ^3^ Department of Neurosurgery, Hulin People’s Hospital, Jixi, Heilongjiang, China; ^4^ Department of Orthopaedic Surgery, Chungnam National University School of Medicine, Daejeon, Republic of Korea; ^5^ School of Nursing, Shandong First Medical University & Shandong Academy of Medical Sciences, Taian, Shandong, China

**Keywords:** high-grade glioma, web-based prognostic nomogram, SEER, overall survival, cancer-specific survival, external validation

## Abstract

**Background:**

Elderly individuals diagnosed with high-grade gliomas frequently experience unfavorable outcomes. We aimed to design two web-based instruments for prognosis to predict overall survival (OS) and cancer-specific survival (CSS), assisting clinical decision-making.

**Methods:**

We scrutinized data from the SEER database on 5,245 elderly patients diagnosed with high-grade glioma between 2000-2020, segmenting them into training (3,672) and validation (1,573) subsets. An additional external validation cohort was obtained from our institution. Prognostic determinants were pinpointed using Cox regression analyses, which facilitated the construction of the nomogram. The nomogram’s predictive precision for OS and CSS was gauged using calibration and ROC curves, the C-index, and decision curve analysis (DCA). Based on risk scores, patients were stratified into high or low-risk categories, and survival disparities were explored.

**Results:**

Using multivariate Cox regression, we identified several prognostic factors for overall survival (OS) and cancer-specific survival (CSS) in elderly patients with high-grade gliomas, including age, tumor location, size, surgical technique, and therapies. Two digital nomograms were formulated anchored on these determinants. For OS, the C-index values in the training, internal, and external validation cohorts were 0.734, 0.729, and 0.701, respectively. We also derived AUC values for 3-, 6-, and 12-month periods. For CSS, the C-index values for the training and validation groups were 0.733 and 0.727, with analogous AUC metrics. The efficacy and clinical relevance of the nomograms were corroborated via ROC curves, calibration plots, and DCA for both cohorts.

**Conclusion:**

Our investigation pinpointed pivotal risk factors in elderly glioma patients, leading to the development of an instrumental prognostic nomogram for OS and CSS. This instrument offers invaluable insights to optimize treatment strategies.

## 1 Introduction

High-grade gliomas rank as the predominant and most virulent primary brain tumors in adults, constituting a significant fraction of malignant gliomas (1). In individuals aged 65 and over, the occurrence of these tumors is 2.63 times that of their younger counterparts (2), presenting amplified challenges due to typically poorer prognoses in this older demographic (3). Given the dire survival statistics, it is imperative to dissect the prognostic factors for overall survival (OS) and cancer-specific survival (CSS) in the elderly to refine clinical decision-making and treatment modalities. Elderly patients with glioma encounter unique challenges compared to their younger counterparts. These challenges include systemic aging, multiple comorbidities which make tolerating the toxic effects of intensive treatments difficult, and a focus on treatment strategies that prioritize improving quality of life. Declines in cognitive and functional status can influence patient compliance, while the surgical risks and incidences of complications and adverse reactions are elevated. For glioma patients, age and overall health status significantly influence prognosis. Despite this, many existing prognostic models for glioma either overlook the nuances of elderly patients or exclude them based solely on age. Such models fail to offer accurate prognostic predictions for individual elderly patients, hindering effective clinical decision-making. Addressing this deficiency, our study aims to develop tailored prognostic assessment tools for the elderly, facilitating personalized outcome predictions and treatment choices.

Clinical and tumor-centric prognostic models can be instrumental in predicting individual risk and outcomes for elderly glioma patients. Nomograms, statistical models that generate personalized probabilities of clinical outcomes like survival based on various predictors (4), have gained traction in oncological decision-making due to their enhanced prognostic precision over conventional staging systems (5). The prognostic nomogram integrates a range of clinical and pathological factors, assigning scores and weights to each based on regression analysis, to quantify a patient’s prognostic risk. Unlike traditional staging systems, nomograms excel in offering individualized, quantitative outcome predictions. By generating risk predictions tailored to a patient’s clinical and pathological profile, they equip physicians with vital insights for devising personalized treatment strategies. For instance, patients with favorable prognoses might be advised to undergo aggressive treatments, including surgery and chemoradiotherapy. Conversely, for those with unfavorable prognoses, considering the potential for tumor progression and complications, a more conservative approach may be recommended to prioritize quality of life. Yet, a conspicuous gap exists in the provision of nomograms specifically calibrated for OS and CSS predictions in elderly patients with high-grade gliomas. While a plethora of prognostic tools populate the academic landscape, only a scant few embrace the convenience and immediacy of web-based solutions. These digital platforms, with their intuitive interfaces, can revolutionize clinicians’ decision-making, ensuring patient-centric, optimal care pathways. In the era of digital health ascendancy, a web-based prognostic tool tailored for this demographic is both timely and essential.

Thus, the crux of our study was twofold: to pinpoint the salient risk factors for elderly patients with high-grade glioma and to architect and validate a web-centric prognostic nomogram for OS and CSS. This nomogram is underpinned by established clinical prognostic markers discerned through multivariate regression analysis from the expansive Surveillance, Epidemiology, and End Results (SEER) database. We envisage that our nomogram will equip healthcare professionals with a tangible, pragmatic instrument to sharpen survival predictions and tailor treatment plans for the elderly glioma cohort. Validation was undertaken with external datasets to enhance its reliability and applicability.

## 2 Methods

### 2.1 Patient selection and data source

Elderly patients with glioblastoma multiforme were identified from the SEER database using SEER*Stat software (Version 8.4.2) through January 2023 (6). We employed the International Classification of Diseases for Oncology, third edition (ICD-O-3) codes to recognize glioblastoma (GBM) cases diagnosed between 2000-2020. The SEER cancer registry, established by the National Cancer Institute in 1973, captures standardized cancer data from diverse U.S. regions, covering 34.6% of the national population. Drawing from hospitals, physicians, laboratories, and vital statistics offices, SEER offers a rich dataset on patient demographics, tumor attributes, treatment, and outcomes. This valuable resource assists in monitoring national cancer statistics, trends, and aids cancer control initiatives. The publicly accessible SEER data facilitates in-depth cancer analysis to guide prevention, treatment, and research strategies. The following variables were extracted for each patient: age (coded as 65 to 69 years, 70 to 69 years) 79 or ≥80 years), sex, race (white, black, or other), marital status (married, unmarried, or other), tumor grade (class III or IV), primary tumor site (supratentorial, cerebellum/brainstem, overlap area, or unspecified), laterality (left, right, or other), and tumor size (<4.5 cm or ≥4.5 cm) cm), extent of lesion (localized, regional, or distant), type of surgery (none, subtotal, or total resection), and use of radiotherapy and chemotherapy (yes or no/unknown). We chose these variables because previous studies have shown that they may be prognostic factors for survival outcome in glioma patients. Age, extent of resection, and modalities such as radiotherapy and chemotherapy have long been considered important determinants of prognosis. Characteristics such as tumor location, size, and grade can also significantly affect clinical outcomes.

The ICD-O-3, crafted by the World Health Organization, ensures precise classification of neoplasms based on anatomy and histology. It promotes standardization across over 1500 histological types, using four-digit codes for location and two-digit codes for microscopic composition. By ensuring consistent tumor categorization, the regularly updated ICD-O-3 bolsters cancer surveillance and research, enabling comparison of national and global incidence data. Given the SEER dataset’s public accessibility, there was no need for ethics committee approval or informed consent.

Our primary focus was on high-grade gliomas in elderly patients. The inclusion criteria were:

(1) First or primary malignant glioma, excluding other primary cancers;
(2) Diagnostic confirmation by positive histology;
(3) Grade III-IV glioma, excluding unclassified cases;
(4) Age ≥ 65;
(5) Predominant histological types of high-grade gliomas listed by specific codes;
(6) Exclusion of ambiguous or invalid primary tumor dimensions;
(7) Surgical type specifications, excluding unknown or diagnostic surgeries;
(8) Excluding unknown or unspecified laterality records;
(9) Exclusion of patients with unspecified demographic details.

**Figure 1:**
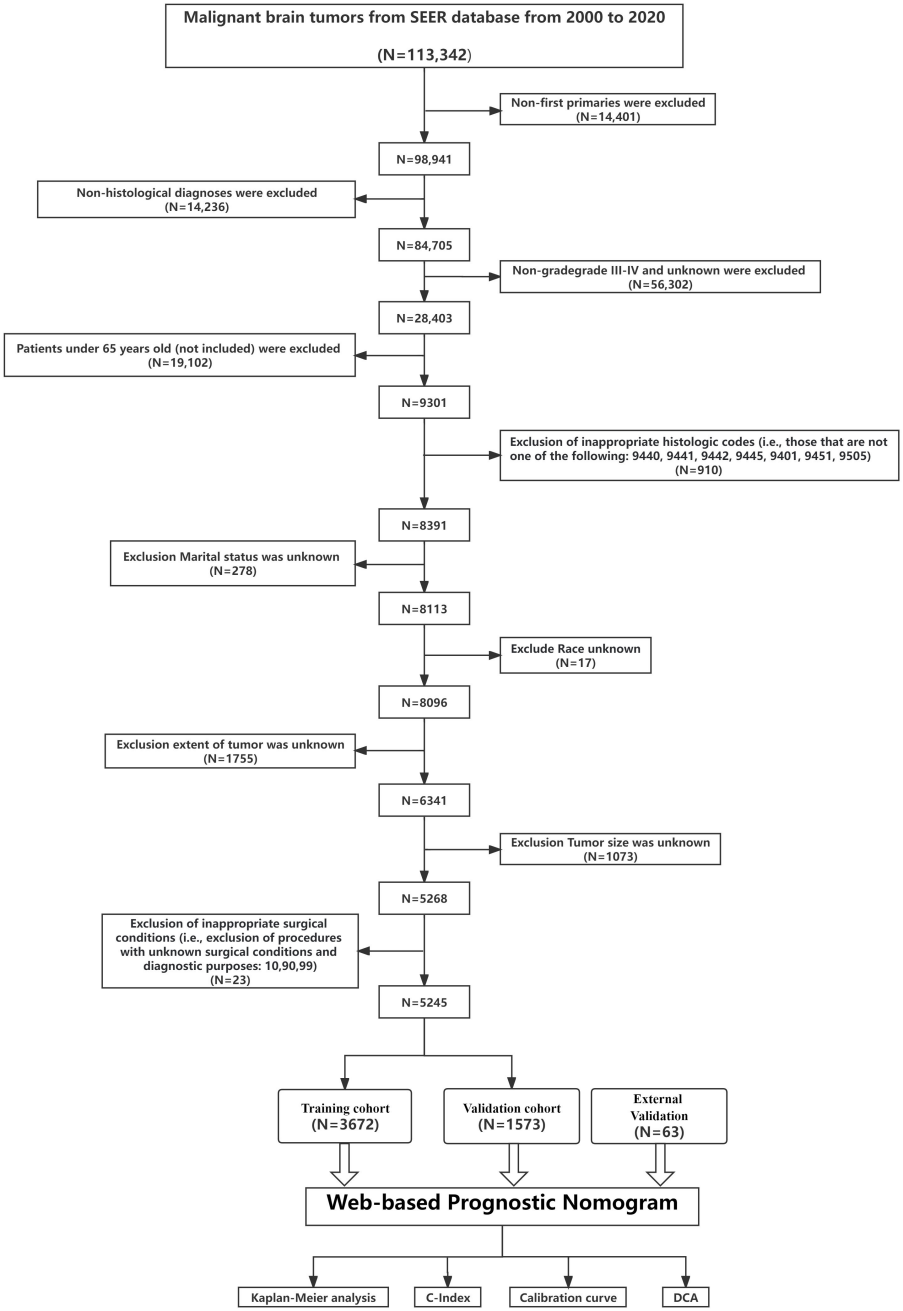
Overview of the study. The flowchart illustrates the step-by-step progression from Data extracted from the SEER database to Validation of the prediction model. Each box represents a distinct phase, interconnected by arrows indicating the flow or sequence. C-index, concordance index; SEER, Surveillance, Epidemiology, and End Results; ROC, receiver operating characteristic; DCA, decision curve analysis.

For external validation, we retrospectively sourced data from elderly high-grade glioma patients at the Fourth Affiliated Hospital of Harbin Medical University and Hulin People’s Hospital between 2008-2023. This external cohort’s inclusion and exclusion criteria mirrored the primary SEER dataset. All participants from the external validation group provided informed consent. The study received local ethics committee approval and conformed to the Declaration of Helsinki. **Figure 1** depicts the patient selection flow. From our screening, 5245 glioma patients were shortlisted and randomly segmented into training (3672) and internal validation (1573) cohorts, with an additional external validation cohort of 63 patients.

### 2.2 Variables and definitions

We extracted twelve attributes from the SEER database, deemed potentially prognostic for prostate cancer patients with brain metastases. The profile of geriatric glioblastoma patients encompassed the following factors: demographics (including age, sex, race, and marital status), tumor characteristics (such as grade, primary site, laterality, size, and extent of spread), and treatment modalities (surgery type, radiotherapy, and chemotherapy). Each variable was categorized based on SEER database codes: Age: 65-69, 70-79, or ≥80 years; Race: White, Black, or other (comprising American Indian/Alaska Native and Asian/Pacific Islander); Marital Status: Married, unmarried (which includes single or domestic partner), or other (encompassing separated, divorced, or widowed); Tumor Size: Less than 4.5 cm or equal to/greater than 4.5 cm; Laterality: Left, right, or other (indications like not paired, bilateral, or midline); Primary Site: Supratentorial lobes, cerebellum/brainstem, overlapping regions, or unspecified; Extent (or summary stage): Local, regional, or distant; Surgery: None, gross total resection, or subtotal resection. By categorizing age and tumor size into distinct groups, it becomes more straightforward for clinicians to gauge risk profiles and potentially tailor interventions based on these simpler categorizations. Our categorization was grounded in clinically established thresholds or prior research.

### 2.3 Cox regression and nomogram development

In the training cohort, potential prognostic factors were ascertained using univariate Cox regression analysis. Those with a *P*-value less than 0.05 in the univariate analysis underwent multivariate Cox regression to identify independent prognostic determinants. A prognostic nomogram was then formulated for predicting OS and CSS based on these independent factors. “ Hazard Ratio (HR)” is used to denote hazard ratios derived from the Cox regression analyses.

Model discrimination and performance were evaluated using Harrell’s concordance index (C-index) and receiver operating characteristic (ROC) curves. The area under the curve (AUC) was calculated for the ROC curves to gauge the model’s accuracy (7). C-index and AUC values ranging between 0.5 and 1.0 signify better predictive precision. The R package “rms” facilitated the generation of calibration plots, providing insight into the nomogram’s accuracy.

For assessing the clinical relevance of the nomogram, decision curve analysis (DCA) was deployed (8). Upon identifying the optimal cutoff for risk scores, a risk stratification system was established. Based on this demarcation, patients in both cohorts were classified into high-risk or low-risk categories. Kaplan-Meier curves and log-rank tests were employed to discern survival differences between these risk groups.

The nomogram development process can be summarized as follows: 1. Assignment of Points for Each Variable: Points for each variable were determined using regression coefficients from our multivariable Cox proportional hazards model. A unit increase in a predictor variable leads to a proportional increase in the log hazard ratio, and thus risk, as defined by its regression coefficient. Setting one predictor (usually with the smallest coefficient) as a reference (e.g., 100 points) allowed for the scaling of other predictors’ coefficients in relation to this benchmark, establishing their respective point values; 2. Rationale Behind Point Assignments: This method of point allocation provides a graphical simplification of the complex mathematical interplay between predictors and outcomes, aiding clinicians and researchers in calculating an aggregate point score that denotes a patient’s specific risk or likelihood of an outcome; 3. Translating Total Points to Survival Probabilities: Aggregate points from predictors were linked to survival probabilities using the baseline survival function. The survival probability corresponding to a particular point score was determined by integrating the score into our cohort’s derived baseline survival function.

In this study, point scores in the nomogram were assigned based on the β-coefficients obtained from the Cox regression models. The prognostic factor with the largest absolute β-coefficient was allocated a score of 100 points. Subsequent prognostic factors were scored relative to this benchmark, according to their individual β-coefficients. There were no additional modifications or adjustments to the β-coefficients beyond this relative scoring process. Using these assigned point scores, the nomogram was developed by aligning each prognostic determinant with its corresponding point range. The cumulative points from all determinants were then mapped to the predicted probabilities of OS and CSS on the nomogram’s outcome axis.

### 2.4 Statistical analysis

All statistical analyses were performed using R software (version 4.1.3). Continuous variables, such as OS presented in months, are depicted as medians with interquartile ranges (Q1, Q3). Categorical variables are conveyed through frequencies and percentages. Chi-squared tests evaluated categorical variables, whereas t-tests analyzed continuous variables. Kaplan-Meier curves, constructed to assess survival rates, were compared using log-rank tests. To discern independent prognostic factors, both univariate and multivariate Cox regression analyses were executed. R packages, including “survival”, “rms”, “timeROC”, “ggplot2”, “ggDCA”, and “DynNom”, facilitated the development, evaluation, and web-based deployment of the prognostic nomogram models. A *P*-value less than 0.05 (two-sided) was deemed statistically significant.

## 3 Results

### 3.1 Characteristics of baseline cohort

In this study, 5,245 elderly patients diagnosed with high-grade glioma were selected from the SEER database based on specific inclusion and exclusion criteria. These patients were randomly divided into a training cohort (n = 3,672) and an internal validation cohort (n = 1,573) using a 7:3 ratio. Additionally, 63 elderly patients with high-grade glioma from the Fourth Affiliated Hospital of Harbin Medical University and Hulin People’s Hospital were included as an external validation cohort, comprising 20 patients aged 65-69, 31 aged 70-79, and 12 aged 80 and above.

**Table 1** presents the baseline clinicopathological attributes of the participants. It’s noteworthy that 48.5% of the patients were aged between 70-79 years, with a significant majority (over 90%) being white. Most had grade IV gliomas (over 90%). About 70% exhibited gliomas situated in the supratentorial lobes, and over 90% had tumors with localized extent. More than half of the patients (53.4%) had tumors smaller than 4.5 cm. In terms of tumor laterality, 42.6% were on the left side, and 43.1% on the right. Additionally, over 60% of the patients underwent both chemotherapy and radiotherapy.

**Table 1:** Characteristics of elderly patients with high-grade glioma.

Variables	Total (n = 5245)	Training cohort	Validation cohort	*P*
		(n = 3672)	(n = 1573)	
**Age**				0.937
65-69	1806 (34.4%)	1270 (34.6%)	536 (34.1%)	
70-79	2545 (48.5%)	1777 (48.4%)	768 (48.8%)	
≥80	894 (17.0%)	625 (17.0%)	269 (17.1%)	
**Sex**				0.596
Female	2415 (46.0%)	1700 (46.3%)	715 (45.5%)	
Male	2830 (54.0%)	1972 (53.7%)	858 (54.5%)	
**Race**				0.815
White	4779 (91.1%)	3351 (91.3%)	1428 (90.8%)	
Black	230 (4.4%)	160 (4.4%)	70 (4.5%)	
Other	236 (4.5%)	161 (4.4%)	75 (4.8%)	
**Marital Status**				0.810
Unmarried	455 (8.7%)	324 (8.8%)	131 (8.3%)	
Married	3468 (66.1%)	2428 (66.1%)	1040 (66.1%)	
Other	1322 (25.2%)	920 (25.1%)	402 (25.6%)	
**Grade**				0.250
III	350 (6.7%)	235 (6.4%)	115 (7.3%)	
IV	4895 (93.3%)	3437 (93.6%)	1458 (92.7%)	
**Primary Site of Glioma**				0.995
Supratentorial lobes	4111 (78.4%)	2879 (78.4%)	1232 (78.3%)	
Cerebellum and brainstem	41 (0.8%)	28 (0.8%)	13 (0.8%)	
Overlapping	761 (14.5%)	532 (14.5%)	229 (14.6%)	
Unspecified location	332 (6.3%)	233 (6.3%)	99 (6.3%)	
**Laterality**				0.781
Left	2236 (42.6%)	1554 (42.3%)	682 (43.4%)	
Right	2263 (43.1%)	1594 (43.4%)	669 (42.5%)	
Other	746 (14.2%)	524 (14.3%)	222 (14.1%)	
**Extent of glioma**				0.872
Localized	4214 (80.3%)	2944 (80.2%)	1270 (80.7%)	
Regional	939 (17.9%)	664 (18.1%)	275 (17.5%)	
Distant	92 (1.8%)	64 (1.7%)	28 (1.8%)	
**Tumor Size (cm)**				0.069
<4.5	2799 (53.4%)	1929 (52.5%)	870 (55.3%)	
≥4.5	2446 (46.6%)	1743 (47.5%)	703 (44.7%)	
**Surgery**				0.442
No	1468 (28.0%)	1010 (27.5%)	458 (29.1%)	
Subtotal Resection	1787 (34.1%)	1266 (34.5%)	521 (33.1%)	
Gross Total Resection	1990 (37.9%)	1396 (38.0%)	594 (37.8%)	
**Chemotherapy**				0.621
No/Unknown	2029 (38.7%)	1412 (38.5%)	617 (39.2%)	
Yes	3216 (61.3%)	2260 (61.5%)	956 (60.8%)	
**Radiotherapy**				0.343
No/Unknown	1584 (30.2%)	1094 (29.8%)	490 (31.2%)	
Yes	3661 (69.8%)	2578 (70.2%)	1083 (68.8%)	
**OS (months)** Median (Q1, Q3)	5 (2, 12)	5 (2, 12)	5 (2, 11)	0.051
**Status**				0.882
Alive	500 (9.5%)	352 (9.6%)	148 (9.4%)	
Dead	4745 (90.5%)	3320 (90.4%)	1425 (90.6%)	

OS, overall survival.

### 3.2 Identification of prognostic factors

Univariate Cox regression analysis was performed on all variables within the training cohort to discern factors influencing overall survival. Variables significant at a *P*-value less than 0.05 included age, marital status, glioma’s primary site, laterality, glioma extent, tumor size, surgical intervention, chemotherapy, and radiotherapy. These variables were subsequently incorporated into the multivariate Cox regression model. Upon multivariate analysis, age, glioma’s primary site, laterality, glioma extent, tumor size, surgical approach, chemotherapy, and radiotherapy remained statistically significant predictors of overall survival for elderly patients with high-grade glioma, with *P*-values of <0.001, 0.016, <0.001, <0.001, 0.013, <0.001, <0.001, and <0.001, respectively. Notably, for CSS, significant factors included age, glioma’s primary site, laterality, glioma extent, tumor size, surgical approach, chemotherapy, and radiotherapy, with corresponding *P*-values of <0.001, 0.022, <0.001, <0.001, 0.011, <0.001, <0.001, and <0.001. Collectively, these findings underscore that patient age, glioma location, laterality, extent, size, and treatment modalities significantly determine survival outcomes in this patient demographic (refer to **Table 2** for details).

**Table 2:** Analyses of overall survival and cancer-specific survival in elderly patients with high-grade glioma using both univariate and multivariate regression.

Subject Characteristics	Overall Survival						Cancer-Specific Survival					
	Univariate analysis			Multivariate analysis			Univariate analysis			Multivariate analysis		
	HR	95% CI	*P*	HR	95% CI	*P*	HR	95% CI	*P*	HR	95% CI	*P*
Age												
65-69	ref			ref			ref			ref		
70-79	1.31	1.22-1.42	<0.001	1.24	1.15-1.34	<0.001	1.29	1.19-1.40	<0.001	1.21	1.12-1.31	<0.001
≥80	2.34	2.12-2.59	<0.001	1.66	1.49-1.85	<0.001	2.32	2.09-2.57	<0.001	1.64	1.47-1.83	<0.001
Sex												
Female	ref						ref					
Male	1.05	0.98-1.12	0.190				1.03	0.96-1.11	0.376			
Race												
White	ref						ref					
Black	0.98	0.83-1.16	0.777				0.94	0.79-1.12	0.476			
Other	0.87	0.73-1.03	0.115				0.86	0.72-1.03	0.098			
Marital Status												
Unmarried	ref			ref			ref			ref		
Married	0.85	0.76-0.96	0.011	1.01	0.90-1.15	0.846	0.88	0.77-1.00	0.045	1.04	0.92-1.19	0.519
Other	1.13	0.99-1.29	0.071	1.04	0.91-1.19	0.537	1.15	1.00-1.32	0.052	1.06	0.92-1.22	0.404
Grade												
III	ref						ref					
IV	0.98	0.85-1.13	0.793				1.01	0.87-1.18	0.856			
Primary Site of Glioma												
Supratentorial lobes	ref			ref			ref			ref		
Cerebellum and brainstem	1.53	1.05-2.22	0.025	1.58	1.09-2.30	0.016	1.52	1.03-2.24	0.033	1.57	1.07-2.32	0.022
Overlapping	1.01	0.92-1.11	0.857	1.05	0.95-1.15	0.368	0.98	0.88-1.08	0.648	1.01	0.91-1.12	0.841
Unspecified location	1.13	0.98-1.30	0.105	1.11	0.96-1.28	0.158	1.10	0.95-1.28	0.193	1.09	0.94-1.26	0.271
Laterality												
Left	ref			ref			ref			ref		
Right	1.05	0.97-1.13	0.209	1.05	0.97-1.13	0.206	1.06	0.98-1.14	0.159	1.06	0.98-1.14	0.155
Other	1.46	1.32-1.62	<0.001	1.24	1.12-1.38	<0.001	1.47	1.32-1.64	<0.001	1.25	1.12-1.40	<0.001
Extent of glioma												
Localized	ref			ref			ref			ref		
Regional	1.43	1.31-1.56	<0.001	1.27	1.16-1.40	<0.001	1.42	1.30-1.56	<0.001	1.26	1.14-1.40	<0.001
Distant	1.6	1.24-2.06	<0.001	1.51	1.17-1.95	0.002	1.63	1.26-2.11	<0.001	1.53	1.18-2.00	0.001
Tumor Size												
<4.5	ref			ref			ref			ref		
≥4.5	1.1	1.03-1.18	0.005	1.09	1.02-1.17	0.013	1.11	1.03-1.19	0.006	1.10	1.02-1.18	0.011
Surgery												
No	ref			ref			ref			ref		
Subtotal Resection	0.56	0.51-0.61	<0.001	0.65	0.60-0.71	<0.001	0.55	0.50-0.60	<0.001	0.64	0.58-0.70	<0.001
Gross Total Resection	0.47	0.43-0.51	<0.001	0.56	0.51-0.61	<0.001	0.46	0.42-0.50	<0.001	0.55	0.50-0.61	<0.001
Chemotherapy												
No/Unknown	ref			ref			ref			ref		
Yes	0.38	0.35-0.40	<0.001	0.6	0.55-0.66	<0.001	0.37	0.35-0.40	<0.001	0.60	0.55-0.66	<0.001
Radiotherapy												
No/Unknown	ref			ref			ref			ref		
Yes	0.32	0.29-0.34	<0.001	0.48	0.43-0.52	<0.001	0.32	0.29-0.34	<0.001	0.48	0.43-0.53	<0.001

HR, hazard ratio; CI, confidence interval.

### 3.3 Development and validation of the prognostic nomogram

**Figure 2:**
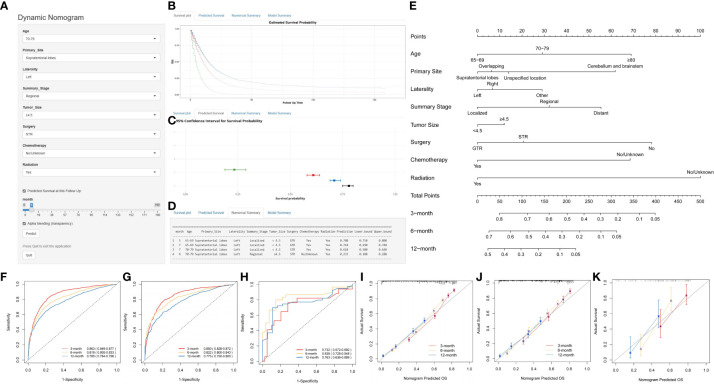
Development and Validation of a Web-Based Nomogram to Predict 3-, 6-, and 12-Month Overall Survival in Elderly Patients with High-Grade Glioma. The web-based nomogram on overall survival **(A)**. Curve depicting estimated survival probability over time for the input patient **(B)**. 95% confidence intervals for selected predicted monthly survival probabilities **(C)**. Numerical summary of predicted monthly survival probabilities **(D)**. Nomogram on overall survival in elderly patients with high-grade glioma **(E)**. ROC curves in the training group **(F)**, the internal validation group **(G)** and external validation **(H)**. Calibration curves were generated for the training cohort **(I)**, the internal validation cohort **(J)** and the external validation **(K)**. User guide for the nomogram: For each patient, a vertical line from each variable value intersects the “Points” axis to determine its score. The cumulative score is determined based on the axis labeled as ‘Total Points’. Next, a vertical line is drawn downwards from the sum of points to determine the predicted overall survival of 3-, 6-, and 12-month. User guide for the web-based nomogram: Log on to the website, enter the age, primary site, laterality, summary stage, tumor size, surgery, chemotherapy, and radiation into the line according to the actual situation of the patient, select predicted survival n months, and then click “Predict”. If high traffic prevents normal use, click “Reload” in the bottom left corner to retry. STR, subtotal resection; GTR, gross total resection.

**Figure 3:**
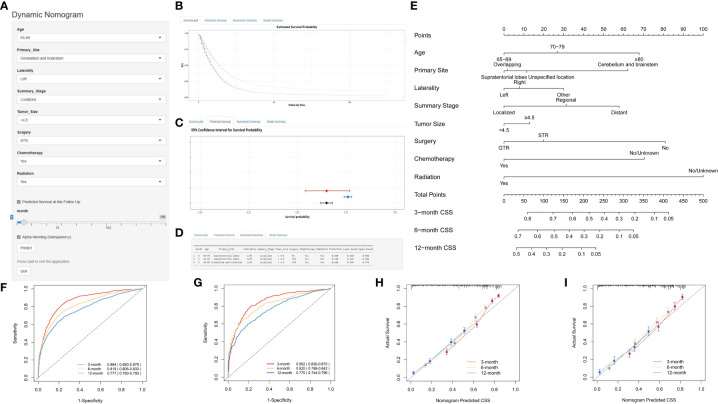
Development and Validation of a Web-Based Nomogram for Predicting 3-, 6-, and 12-Month Cancer-Specific Survival in Elderly Patients with High-Grade Glioma. The web-based nomogram on cancer-specific survival **(A)**. Curve depicting estimated survival probability over time for the input patient **(B)**. 95% confidence intervals for selected predicted monthly survival probabilities **(C)**. Numerical summary of predicted monthly survival probabilities **(D)**. Nomogram on overall survival in elderly patients with high-grade glioma **(E)**. ROC curves in the training group **(F)** and validation group **(G)**. Calibration curves were generated for the training cohort **(H)** and the validation cohort **(I)**. The User guide is the same as in **Figure 2**.

Using multivariate Cox regression analysis, eight independent risk factors were identified, and a nomogram was constructed to predict 3-, 6-, and 12-month OS and CSS in elderly patients with high-grade glioma (**Figures 2E** and **3E**). Each variable was assigned a score from 0 to 100 based on its prognostic significance. The combined score, calculated from the sum of individual variable scores, reflected the projected 3-, 6-, and 12-month survival rates. Calibration curves revealed a strong alignment between the nomogram predictions and observed outcomes at 3, 6, and 12 months for both the training and internal validation cohorts, underscoring the nomogram’s high predictive accuracy (**Figures 2I–K**, **3H, I**). The C-index for OS was 0.734 (95% CI: 0.725–0.743) in the training cohort, 0.729 (95% CI: 0.715–0.743) in the internal validation cohort, and 0.701 (0.620-0.781) in the external validation cohort. AUC values for these cohorts were as follows: for the training cohort, they were 0.863 at 3 months, 0.819 at 6 months, and 0.780 at 12 months (**Figure 2F**); for the internal validation cohort, they were 0.850 at 3 months, 0.822 at 6 months, and 0.775 at 12 months (**Figure 2G**); for the external validation cohort, they were 0.732 at 3 months, 0.838 at 6 months, and 0.763 at 12 months (**Figure 2H**). These metrics exhibit robust discriminative capacity, reinforcing the nomogram’s predictive precision. Similarly, the C-index for CSS was 0.733 (95% CI: 0.724-0.742) in the training cohort and 0.727 (95% CI: 0.713-0.741) in the validation cohort. The associated AUC values were 0.864 at 3 months, 0.819 at 6 months, and 0.777 at 12 months (**Figure 3F**) in the training cohort, and 0.852 at 3 months, 0.820 at 6 months, and 0.770 at 12 months (**Figure 3G**) in the validation cohort. These metrics also showcase strong discriminative power, further affirming the nomogram’s predictive accuracy. In summary, the proposed nomogram presents a reliable method for individualized outcome prediction in elderly patients with high-grade glioma.

### 3.4 Clinical application of the nomogram

**Figure 4:**
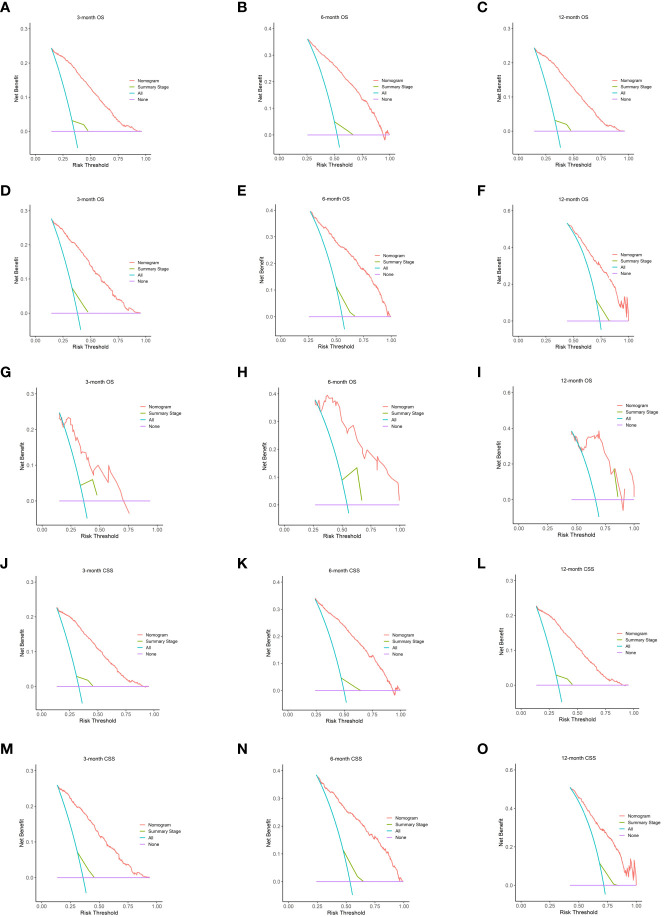
The DCA of the nomogram. On OS for predicting 3-month **(A)**, 6-month **(B)**, and 12-month **(C)** in the training cohort; 3-month **(D)**, 6-month **(E)**, and 12-month **(F)** in the internal validation cohort and 3-month **(G)**, 6-month **(H)**, and 12-month **(I)** in the external validation cohort. The DCA of the nomogram on CSS for predicting 3-month **(J)**, 6-month **(K)**, and 12-month **(L)** in the training cohort and 3-month **(M)**, 6-month **(N)**, and 12-month **(O)** in the validation cohort. Summary stage is equal to extent of glioma. DCA, decision curves analysis; OS, overall survival; CSS, cancer-specific survival.

We assessed the utility of our nomogram against the summary stage using decision curve analysis. This analysis demonstrated that our nomogram consistently provided a higher net clinical benefit, producing more precise 3-, 6-, and 12-month OS and CSS predictions compared to the summary stage. The external validation cohort further validated this advantage, underscoring the clinical efficacy of our nomogram (**Figure 4**).

To enhance the nomogram’s clinical applicability, we developed an intuitive point scale for straightforward bedside use. As illustrated in **Figures 2E** and **3E**, physicians can align a patient’s prognostic indicators with the corresponding points. By summing the total points and referencing the total point scale, clinicians can directly ascertain the projected 3-, 6-, and 12-month OS and CSS. For each patient, a vertical line drawn from the variable value intersects the ‘Points’ axis to determine the corresponding score. The combined score is inferred from the ‘Total Points’ axis, and another vertical line from this total score indicates the predicted OS and CSS for 3, 6, and 12 months. This streamlined point system effortlessly merges the nomogram into clinical routines, offering tailored survival predictions that can inform patient discussions and treatment decisions tailored to the risks for elderly glioma patients. Parameters such as the extent of resection can be adaptively modified to refresh prognostic estimates during patient follow-ups.

### 3.5 Application of risk stratification system

**Figure 5:**
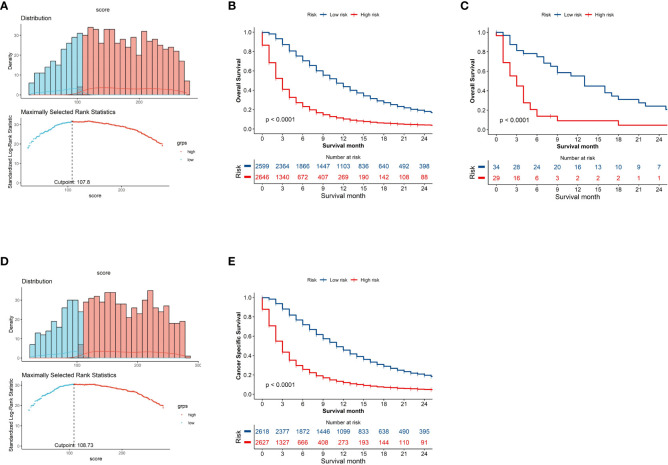
Kaplan–Meier curves demonstrating Overall Survival (OS) and Cancer-Specific Survival (CSS) in low- and high-risk patient groups. The x-axis represents time and the y-axis shows the probability of survival. The drops in the curve represent observed events (deaths) at that time point. Histogram depicting distribution of patients based on optimal risk score cut-off point determined by X-tile software on OS **(A)** and CSS **(D)**. Kaplan-Meier curves demonstrating SEER cohort **(B)** and the external validation **(C)** on OS and SEER cohort on CSS **(E)** in low- and high-risk groups.

X-tile employs a data-driven approach complemented by statistical simulations and modeling to determine optimal cut point for biomarkers that maximize sensitivity and specificity for outcomes such as survival (9). The implemented algorithms include equal-width binning, equal-frequency binning, optimal data-driven binning, Monte Carlo simulations, Kaplan-Meier analysis, and bootstrapping (10). This rigorous approach enables optimal biomarker cut point determination and has led to the frequent utilization of X-tile for survival analysis across various malignant tumors (11–13). In this study, the X-tile algorithms enabled reliable optimal cut-point analysis and creation of survival-based risk stratification systems using nomogram scores for all patients. The entire cohort was divided into two distinct risk subgroups: low-risk (N = 2599, 49.55%, scores <107.8) and high-risk (N = 2646, 50.45%, scores >107.8) on OS (**Figure 5A**), which displayed substantial differences in Kaplan-Meier survival curves, validating the risk stratification system. A similar stratification was observed when the cohort was divided into low-risk (N = 2618, 49.91%, scores <108.73) and high-risk (N = 2627, 50.09%, scores >108.73) subgroups on CSS (**Figure 5D**), which also exhibited significant differences in Kaplan-Meier survival curves, further corroborating the validity of the risk stratification system. Analysis of survival using Kaplan-Meier curves and log-rank tests indicated that the subgroup at high risk exhibited decreased survival rates in comparison to the low-risk subgroup (**Figure 5B, C, E**).

### 3.6 Web-based nomogram

Web-based nomograms are interactive online prognostic tools that incorporate important predictive factors into graphical calculating devices to provide individualized and precise outcome predictions, beyond traditional staging systems, to guide clinical decision-making. Developed from multivariate analyses of datasets, nomograms allow users to obtain personally tailored risk assessments by entering patient parameters. Their user-friendly web interface facilitates dissemination and validation across clinical settings to aid evidence-based, personalized treatment decisions and counselling regarding recurrence risks, survival outcomes, or post-treatment complications. A user-friendly, web-based dynamic nomogram was developed that physicians and patients could access from any electronic device. As shown in **Figures 2A–D** and **3A–D**, the web-based nomogram allows doctors and patients to input common clinical variables to visually assess individualized postoperative OS (https://prenom.shinyapps.io/DynNomapp_Glioma/) and CSS (https://prenom.shinyapps.io/DynNomapp_CSS/) for elderly patients with high-grade glioma. The legend demonstrates the specific usage method.

## 4 Discussion

Clinical management of elderly patients with high-grade glioma is challenging given their frail health, multiple comorbidities, and heightened sensitivity to chemoradiotherapy toxicity (14). Most clinical studies, including randomized controlled trials (RCTs), often exclude elderly patients with high-grade glioma, leading to an absence of clear treatment guidelines and prognostic models for this demographic. In this study, we developed a prognostic scoring system based on multivariate analysis to provide individualized survival assessment and risk stratification for elderly patients with high-grade gliomas. By retrospectively analyzing data from 5,245 elderly patients in the SEER database, a comprehensive national cancer database, we found that age, primary tumor site, tumor laterality, tumor extent, tumor size, surgery, chemotherapy, and radiotherapy were independent prognostic factors. Based on these factors, we developed two web-based online prognostic scoring systems that can predict individualized survival rates based on patients’ clinical characteristics. Our study provides a valuable tool for prognostic evaluation and risk stratification in elderly patients with high-grade gliomas.

Building upon existing literature, this study also had unique features compared to prior prognostic models developed for high-grade gliomas. A key novel aspect was the creation of an online, individualized prognostic scoring system, different from many previous glioma prognostic tools utilize traditional scoring systems or are not web-accessible for immediate point-of-care use (15, 16). Our user-friendly nomogram provides a practical and comprehensive tool for clinicians to obtain real-time survival predictions tailored to individual patients’ profiles. Compared to other similar nomograms, our study incorporated additional prognostic factors shown to be relevant in elderly glioma patients, including precise tumor location and lateralization rather than only broad categories (e.g. supra- vs infratentorial) (17, 18). However, consistent with previous findings, we also identified age and treatment modalities as significant independent predictors of survival (15).

In comparison to prior studies on the prognosis of elderly glioblastoma patients (19), our study demonstrated a moderately higher C-Index for both OS and CSS. For OS, the C-index in our training cohort was 0.734, compared to 0.715 in previous studies, and 0.729 versus 0.726 in the validation cohort. Similarly, for CSS, the C-Index in our training cohort was 0.733, compared to 0.700 in earlier research, and 0.727 versus 0.707 in the validation cohort. We refined the classification of the primary glioma site into four categories: supratentorial lobes, cerebellum and brainstem, overlapping regions, and unspecified locations. A more detailed classification of the primary glioma site enhances the predictive accuracy of the model. Our study broadened the scope by focusing on elderly patients with a range of high-grade gliomas (WHO III-IV), enhancing the clinical relevance and applicability of our findings. Unlike previous studies that primarily focused on glioblastoma, we incorporated a wider variety of high-grade glioma types, including glioblastoma, giant cell glioblastoma, gliosarcoma, glioblastoma (IDH-mutant), astrocytoma anaplastic, oligodendroglioma anaplastic, and ganglioglioma anaplastic. This comprehensive inclusion improved the predictive accuracy of our model.

Different from the previous research (19), our study did not identify race as an independent predictor for either OS or CSS. This discrepancy might be attributed to differences in sample size and study time points. Moreover, the primary site of glioma emerged as an independent predictor for both OS and CSS in our analysis. This distinction may arise from our study’s more granular classification, wherein the primary site of glioma was categorized into four groups as said above, thereby amplifying its prognostic impact. Additionally, our research recognized tumor extent as an independent predictor for both OS and CSS. We classified the extent of glioma into three categories: Localized, Regional, and Distant. ‘Localized’ denotes a tumor confined to its primary site without distant metastasis. ‘Regional’ signifies tumor invasion into surrounding tissues or regional lymph nodes without distant metastasis, while ‘Distant’ indicates tumors with distant metastases, such as in the cerebrospinal fluid, ventricles, or other body parts. This detailed classification enhances the predictive precision of our model. Consistent with the previous results (20), univariate and multivariate Cox regression analyses identified six prognostic factors: tumor site, laterality, histological type, extent of surgery, radiotherapy, and chemotherapy.

The presence of comorbidities and concerns regarding treatment toxicity may contribute to elderly patients with high-grade glioma declining active therapy after diagnosis, leading to poorer survival prognoses (21). While surgery, radiotherapy, and chemotherapy are standard treatments for glioma, there is no consensus on the optimal approach for elderly patients with high-grade gliomas, as most clinical trials have excluded this older demographic (22). Due to the infiltrative growth pattern, total resection of gliomas is challenging. However, maximal safe surgical resection has been associated with improved prognosis in patients with high-grade gliomas, including elderly populations (23). Importantly, radiotherapy and chemotherapy may improve survival despite not directly improving general condition or quality of life. Treatment side effects should be weighed against potential survival benefit (24). The Web-based nomograms provide individualized risk assessment that can inform discussions around treatment intensity, such as whether aggressive multi-modality treatment is likely to provide meaningful survival benefit or if more conservative options may be more appropriate considering the patient’s predicted prognosis.

Consistent with previous studies (25), tumor extent (local or distant) and metastasis are important prognostic factors in gliomas, with patients having distant or metastatic disease demonstrating poorer survival prognosis. Local invasion or distant metastasis of gliomas has consistently been a key factor impacting prognosis. Studies have shown that gliomas with metastases tend to have a poor prognosis (26, 27). The presence of distant metastases signifies that tumor cells have disseminated via vasculature or cerebrospinal fluid, indicative of advanced disease with heightened treatment challenge. Hence, distant metastasis represents a pivotal parameter for gauging malignancy grade and prognosis in the clinical staging of gliomas.

This study demonstrates poor prognosis for gliomas located in the cerebellum and brainstem, consistent with previous studies (28, 29), maybe attributable to surgical challenges, disruption of critical functional regions, heightened tumor invasiveness, increased postoperative complications, and reduced efficacy of adjuvant therapies. The cerebellum and brainstem comprise critical functional hubs, conferring substantial surgical risks that often preclude total tumor resection. Residual neoplastic cells readily facilitate relapse and progression. As the cerebellum modulates balance and coordination (30) while the brainstem governs respiration and circulation (31), these areas are prone to irreparable neurological impairment from mass effect and operative trauma. Gliomas situated within these sites tend to be higher-grade lesions exhibiting robust invasive and regenerative potential, with enhanced dissemination and metastatic spread. Resection of such cerebellar and brainstem gliomas confers heightened surgical hazards, with increased postoperative complications like cerebral edema and infection that directly jeopardize patient survival.

Our external validation cohort comprised 63 patients from our institution. While smaller than the primary dataset from the SEER database, this cohort included all eligible patients available during the study period. The smaller sample size may introduce variability in the validation metrics. Specifically, the C-index, which measures discriminative ability, can exhibit instability with smaller samples. A larger cohort would provide more robust and generalizable results. However, even with the smaller size, our external validation provides a preliminary check on the nomogram’s performance in a setting apart from the SEER database. Although the external cohort is smaller, the substantial SEER dataset (1,573 patients) offers confidence in the model’s accuracy and generalizability. We recognize the importance of validating our tools in larger, diverse cohorts. In future studies, we aim to collaborate with other institutions to assemble a larger external validation dataset, further establishing the reliability and generalizability of our nomograms.

Compared to traditional nomogram, advantages of web-based nomogram for analyzing glioma overall survival include: 1) intuitive visualization of prognostic factor effects, 2) straightforward comparisons between groups, 3) multifaceted presentation of results, and user-friendly operation and interpretable outputs. 4) Web-based nomograms clearly depict the distribution and trends in survival time associated with various prognostic factors (e.g. age, grade) and visually convey their impact on prognosis, allows for dynamic risk prediction, offering the ability to update parameters at follow-up. 5) We incorporated a broader range of variables, ensuring a more holistic understanding of factors influencing outcomes in elderly glioma patients. 6) By targeting elderly glioma patients specifically, our model is tailored to this demographic, ensuring higher relevance and accuracy. Juxtaposed nomograms readily facilitate comparison of survival time differences across strata of the same prognostic variable (such as age groups). Beyond survival curves, nomograms can also present median survival times, survival rates, and other statistics for enriched data representation. With simple website-based usage, nomogram output is concise, uncluttered, and readily interpretable.

This study has several notable strengths. This study has developed a robust prognostic nomogram for elderly glioma patients that holds significant clinical implications. First, it provides individualized survival prediction to facilitate patient counseling and personalized treatment recommendations. Patients identified as high-risk could be considered for more aggressive therapies or clinical trials, while low-risk patients may benefit more from less intensive treatment. Second, this nomogram enables risk-based stratification for guiding management strategies. High-risk patients may warrant more frequent imaging surveillance or prophylactic interventions. Low-risk patients could avoid overtreatment and undue harms. Third, the model allows objective risk assessment to optimize clinical trial design. Patients could be assigned to trial arms or adaptive interventions according to their predicted prognosis. This tool supports dynamic risk prediction through the recalibration model with updated parameters. This allows tracking of evolving patient risk profiles over time. With further validation, it holds promise to improve prognostic accuracy, risk stratification, and ultimately, clinical outcomes for elderly glioma patients. Finally, various methods including C-index, AUC, and calibration curves were used to comprehensively validate the predictive performance. Despite the promising results, our study had some limitations that could be addressed in future research. First, prognostic biomarkers such as tumor mutational burden and DNA methylation profiles were not included and may further improve the predictive accuracy. Second, the dynamic change of prognostic factors during treatment and follow-up needs to be examined. Finally, immune status, comorbidities, and other factors that may influence elderly patient prognosis were not incorporated into the scoring system. Based on these limitations, future studies should focus on (1): Incorporation of emerging prognostic biomarkers to enhance individual risk prediction (2). Development of dynamic, longitudinal prognostic models that integrate serial measurements over time (3). Collaboration with other institutions to assemble a larger external validation dataset and establishment the reliability and generalizability of our nomograms.

## 5 Conclusion

Taking advantage of a substantial sample size, this study identified independent prognostic factors for OS and CSS in elderly patients with high-grade glioma and formulated a web-based prognostic nomogram. These nomograms offer predictions on survival probabilities and serves as a clinical reference for treatment strategies and prognosis.

## Data availability statement

The original contributions presented in the study are included in the article/supplementary material. Further inquiries can be directed to the corresponding authors.

## Ethics statement

The studies involving humans were approved by The ethics committee of Hulin People’s Hospital/The ethics committee of the Fourth Affiliated Hospital of Harbin Medical University. The studies were conducted in accordance with the local legislation and institutional requirements. Written informed consent for participation was not required from the participants or the participants’ legal guardians/next of kin in accordance with the national legislation and institutional requirements.

## Author contributions

HZ: Conceptualization, Data curation, Formal Analysis, Writing – original draft. XJ: Investigation, Writing – original draft. FR: Methodology, Writing – original draft. QG: Software, Writing – original draft. JY: Visualization, Writing – original draft. XiW: Validation, Writing – original draft. SZ: Software, Writing – original draft. YG: Data curation, Writing – original draft. JG: Methodology, Writing – original draft. YX: Validation, Writing – original draft. ZW: Investigation, Writing – original draft. SL: Supervision, Writing – review & editing. XW: Resources, Writing – review & editing. BW: Funding acquisition, Writing – review & editing.
